# Tachikawa project for prevention of posttraumatic stress disorder with polyunsaturated fatty acid (TPOP): study protocol for a randomized controlled trial

**DOI:** 10.1186/1471-244X-13-8

**Published:** 2013-01-05

**Authors:** Yutaka Matsuoka, Daisuke Nishi, Naohiro Yonemoto, Kei Hamazaki, Kenta Matsumura, Hiroko Noguchi, Kenji Hashimoto, Tomohito Hamazaki

**Affiliations:** 1 Department of Psychiatry, National Disaster Medical Center, 3256 Midoricho, 190-0014, Tachikawa, Tokyo, Japan; 2 Department of Clinical Epidemiology, Translational Medical Center, National Center of Neurology and Psychiatry, 4-1-1 Ogawahigashi-cho, 187-8551, Kodaira, Tokyo, Japan; 3 National Institute of Mental Health, National Center of Neurology and Psychiatry, 4-1-1 Ogawahigashi-cho, 187-8553, Kodaira, Tokyo, Japan; 4 CREST, Japan Science and Technology Agency, 3256 Midoricho, 190-0014, Tachikawa, Tokyo, Japan; 5 Department of Public Health, Faculty of Medicine, University of Toyama, 2630 Sugitani, 930-0194, Toyama, Toyama, Japan; 6 Division of Clinical Neuroscience, Chiba University Center for Forensic Mental Health, 1-8-1 Inohana, 260-8670, Chuo-ku, Chiba, Japan; 7 Department of Clinical Sciences, Institute of Natural Medicine, University of Toyama, 2630 Sugitani, 930-0194, Toyama, Toyama, Japan

**Keywords:** Fish oil, Omega-3 fatty acid, Docosahexaenoic acid, Eicosapentaenoic acid, Posttraumatic stress disorder, Accidental injury, Prevention

## Abstract

**Background:**

Preclinical and clinical studies suggest that supplementation with omega-3 fatty acids after trauma might reduce subsequent posttraumatic stress disorder (PTSD). To date, we have shown in an open trial that PTSD symptoms in critically injured patients can be reduced by taking omega-3 fatty acids, hypothesized to stimulate hippocampal neurogenesis. The primary aim of the present randomized controlled trial is to examine the efficacy of omega-3 fatty acid supplementation in the secondary prevention of PTSD following accidental injury, as compared with placebo. This paper describes the rationale and protocol of this trial.

**Methods/design:**

The Tachikawa Project for Prevention of Posttraumatic Stress Disorder with Polyunsaturated Fatty Acid (TPOP) is a double-blinded, parallel group, randomized controlled trial to assess whether omega-3 fatty acid supplementation can prevent PTSD symptoms among accident-injured patients consecutively admitted to an intensive care unit. We plan to recruit accident-injured patients and follow them prospectively for 12 weeks. Enrolled patients will be randomized to either the omega-3 fatty acid supplement group (1,470 mg docosahexaenoic acid and 147 mg eicosapentaenoic acid daily) or placebo group. Primary outcome is score on the Clinician-Administered PTSD Scale (CAPS). We will need to randomize 140 injured patients to have 90% power to detect a 10-point difference in mean CAPS scores with omega-3 fatty acid supplementation compared with placebo. Secondary measures are diagnosis of PTSD and major depressive disorder, depressive symptoms, physiologic response in the experiment using script-driven imagery and acoustic stimulation, serum brain-derived neurotrophic factor, health-related quality of life, resilience, and aggression. Analyses will be by intent to treat. The trial was initiated on December 13 2008, with 104 subjects randomized by November 30 2012.

**Discussion:**

This study promises to be the first trial to provide a novel prevention strategy for PTSD among traumatized people.

**Trial registration:**

ClinicalTrials.gov Identifier NCT00671099

## Background

Accidental injury occurs worldwide and accounted for approximately 16% of the world’s burden of disease in 1998 [1]. Advances in injury care systems have since increased the number of seriously injured people who can survive their injuries [2] and severely injured patients might be at higher risk for developing posttraumatic stress disorder (PTSD) [3]. The prevalence of PTSD determined by structured clinical interviews with injured patients consecutively admitted to intensive care units (ICUs) or emergency departments is in the range of 5–30% at 0–3 months after accidental injury [4-10] to 2–23% at 4–12 months after [4-8,10-12]. Comorbidity between PTSD and depressive disorder is also highly prevalent in these injured patients [4,9,13]. Among Japanese patients admitted to the ICU at our hospital after motor vehicle accident (MVA), 8% developed full PTSD, 16% developed partial PTSD, and 23% developed depressive disorder (16% major depressive disorder, 7% minor depressive disorder) at 1 month after accidental injury [9]. This amounts to roughly one in every four patients developing some form of PTSD after MVA. The need for a selective, preventive intervention strategy is therefore clear.

A few studies have reported some positive outcomes in regard to preventing PTSD. These include a randomized controlled trial of cognitive therapy given in the initial months after MVA [14], an open trial of propranolol after accidental injury in a small study population [15], and a randomized controlled trial of propranolol after accidental injury in a small study population [16]. In Japan, however, psychiatric services provided in emergency hospitals are limited [17] and currently fall short of providing adequate and effective psychiatric care.

A growing number of epidemiological studies suggest an association between depression and low dietary intake of omega-3 fatty acids [18-21]. Although several studies have pointed out discrepancies relating to the differential effects of eicosapentaenoic acid (EPA, 20:5*n*-3) versus docosahexaenoic acid (DHA, 22:6*n*-3), previous meta-analyses are supportive of omega-3 fatty acid supplementation in reducing depressive symptoms [22-27]. However, there have been no trials on PTSD conducted thus far. Several biological mechanisms potentially explain the effects of omega-3 fatty acids on psychiatric illness, especially mood and anxiety disorder [22]. Animal studies have revealed that short-term augmentation of dietary omega-3 fatty acids relative to omega-6 fatty acids up-regulated adult neurogenesis [28], and that dietary omega-3 fatty acids elevated levels of brain-derived neurotrophic factor (BDNF), which promotes neuronal survival and growth [29]. In addition, DHA was shown to increase neurite extension and branching of hippocampal neurons in vitro [30] as well as the maturation of neurons and hippocampal neurogenesis in adult rats [31]. Venna at al. showed that the increase in newborn hippocampal cells by polyunsaturated fatty acids occurred in parallel with an increase in hippocampal volume and over-expression of BDNF mRNA and protein in the hippocampus [32].

The hippocampus is crucial for converting short-term memory into long-term memory [33] and can process and temporarily store new memory during the transition period before transferring labile memory to the cortex for permanent storage [34]. In the pathogenesis of PTSD, fear memory becomes excessively consolidated and extinction learning does not progress [35]. Kitamura et al. recently found that the period of hippocampus-dependent fear memory is longer in mice with decreased hippocampal neurogenesis and shorter in mice with active hippocampal neurogenesis [36], indicating that the level of hippocampal neurogenesis is a crucial factor in determining the duration of hippocampal-dependent fear memory. The possibility arises then that the fear memory characteristic to PTSD might be controllable by aptly regulating hippocampal neurogenesis [37].

We hypothesized that promoting adult neurogenesis early in the transition period might facilitate clearance of fear memory in humans [37]. Our preliminary open trial found that post-trial PTSD symptoms were significantly alleviated in injured patients who all took the omega-3 fatty acids compared with historical controls [38,39]. The present study aims to examine the effectiveness of omega-3 fatty acids for preventing PTSD in physically injured patients admitted to the ICU at National Disaster Medical Center, Japan immediately following accidental injury (mostly MVA). This study is called the Tachikawa Project for Prevention of PTSD with Polyunsaturated Fatty Acid (TPOP).

## Methods

### Study design

TPOP is a double-blinded, parallel group, randomized controlled trial that compares an intervention group that receives omega-3 fatty acid supplementation with a parallel control group that receives placebo supplementation. This study was registered at http://clinicaltrials.gov/ct2/show/NCT00671099.

### Participants/eligibility criteria

Participants are solely recruited from the ICU of the National Disaster Medical Center. The following inclusion criteria apply to patients with accidental injury: (1) aged 18 years or older; (2) a native Japanese speaker or non-native speaker with Japanese conversational ability; (3) contact with us within 240 h after injury; and (4) physical and psychological ability to understand the scope of the present trial and to provide written consent for study participation. Exclusion criteria are presence of the following: (1) acute brain parenchymal damage that is obviously irretrievable or subdural or subarachnoidal bleeding detected by computed tomography and/or magnetic resonance imaging; (2) cognitive impairment, defined as a score of <24 on the Mini-Mental State Examination [40]; (3) a serious drinking problem or high *γ-*GTP blood level of ≥100 IU/L on admission; (4) a smoking habit of ≥40 cigarettes per day; (5) history and current suspicion of psychosis or bipolar I disorder; (6) suspicion of alcohol- or capsule-related disorder or eating disorder; (7) serious psychiatric symptoms such as suicidal ideation, self-harm behavior or severe dissociation, or in need of rapid psychiatric treatment; (8) regular treatment with anti-epilepsy medication, lithium, ethyl icosapentate, aspirin, or warfarin within the last 3 months; (9) regular consumption of polyunsaturated fatty acid supplements within the last 3 months; and (10) a habit of eating fish ≥4 times per week, to ensure comparability to patients studied in Western countries.

All participants receive a gift voucher for their participation after each assessment (1,000 JPY [11 USD] during hospitalization, 2,000 JPY [22 USD] after discharge).

### Enrollment procedure

**Figure 1:**
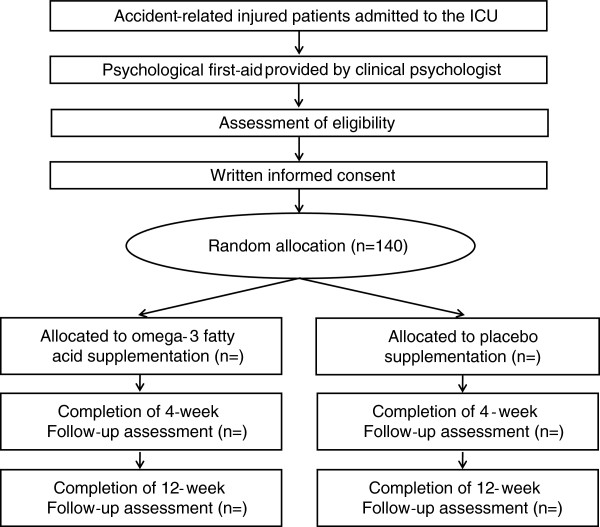
Flow diagram of the study.

Patients receive a comprehensive physical examination including computed tomography or magnetic resonance imaging before the study commences. Eligible patients are screened through the daily conference records for patients newly admitted to the ICU in cooperation with physicians in the Department of Critical Care and Traumatology. From the viewpoint of ethical concerns, all patients are given a self-help leaflet entitled *Understanding Your Reactions to Accidental Injury* before checking the criteria. After receiving a psychological education session for 15–20 min, patients are invited to participate in the study by clinical research coordinators who are well-trained nurses with a Master’s degree in Clinical Psychology. The overall procedure of the trial is shown in Figure 1.

### Informed consent

The following information is used in obtaining consent from eligible participants.

1. Participation in the study is voluntary, and non-participants will not be penalized in any way. Participants can withdraw from the study at any time even after starting participation in the study.

2. The purpose of the study is to examine whether omega-3 capsules are effective in alleviating mental stress after physical trauma compared with placebo capsules in patients with physical trauma transferred to an emergency room.

3. Participants will be randomly assigned to either of the two intervention groups. Participants in both groups will be interviewed at 1 month and 3 months after providing consent.

4. Effectiveness of the intervention will be assessed through interviews as well as testing capsules related to the autonomic nervous systems and biological defense mechanism and neurotrophic factors.

5. Participants surveyed during their hospital stay will receive an incentive item for each interview. Because those who are surveyed after discharge will need to visit the hospital more frequently than usual resulting in increased transportation costs, they will receive incentive items to cover additional transportation costs.

6. Participants will be contacted via telephone, email, or confidential letters. To avoid inconveniencing the participants, their families may be contacted within the agreed conditions.

7. Collected information will be securely stored to protect personal information. Before analysis, identifying information such as name, address, telephone number, date of birth, and patient record number will be removed and new numbers will be assigned (making the data anonymous).

8. All biological samples will be locked in a refrigerator or freezer in the laboratory. They will be stored over the long term because analyses will be conducted after all clinical trials are completed and the materials are needed later for confirmatory experiments.

9. In cases where a new health problem develops during the study, no special compensation will be provided to such participants, but a physician will examine and treat them under universal health insurance.

10. Participants will be immediately provided with information related to the study, including that which may affect their decision to participate in the study.

11. At the request of participants, they can examine any materials such as the research plan, except for other patients’ information.

12. Results from this study may be utilized to improve intervention for mental health treatment for large-scale natural and/or human-made disasters in the future.

13. The study results will be reported by our research team and presented in medical journals and at conferences. However, participants’ personal information will not be revealed.

14. If a patent is granted based on the findings of the study, it will be owned by the principal investigator and the research institute.

15. This study will be conducted with public funding.

16. Participants can contact the investigators if they have any questions.

Informed consent is obtained from eligible patients as follows. Clinical research coordinators first explain the potential public health benefits of the study results as well as the study procedures. Coordinators then explain the study plan, intervention methods, evaluation methods, and means used to obtain information. Eligible patients may take as much time as necessary to assess the information and to decide whether to participate or not. After the study is explained, written documented consent is obtained, and their participation starts (investigators make sure the consent has been filed in the medical record).

During a hospital stay, meetings are regularly held with the participants to ensure they clearly understand some study aspects that lay people may not easily understand, such as the study purpose and treatment assignment. They are asked to strictly adhere to taking the test capsules.

### Intervention

Participants are asked to take 7 capsules per day after eating for 12 weeks and additionally told that they might take a full day’s dosage at one time. Concomitant psychiatric medications are not allowed during the study period except for minimal and intermittent use of hypnotics and antidepressants.

#### Active omega-3 capsule

We prepared new capsules for this trial, containing a higher concentration of DHA than used in our previous trial [41]. Each capsule contains 300 mg of oil with 0.3% alpha-tocopherol. A total of 7 capsules containing 1470 mg DHA and 147 mg EPA are administered daily.

#### Placebo capsule

The content of the control oil was slightly modified from our previous study [42]. The present control oil is a mixture of rapeseed oil (47%), soybean oil (25%), olive oil (25%), fish oil (3%), and 0.3% α-tocopherol. The fatty acid composition of this mixture is similar to the average composition of fatty acid intake in Japan. We added a small amount of not fully deodorized fish oil to the base of the control oil to make it fishy and undistinguishable from the active oil.

#### Management of intervention

During hospitalization, ward nurses administer all capsules and confirm that participants take them. In the case of discharge before completing the trial, participants take the capsules by themselves, and clinical research coordinators telephone or email them to confirm at least once a week. Our weekly e-newsletters are also sent to the participants to maintain their interest in the study and to provide general health care advice. If participants forget to take capsules, they are asked to take a double dose on the next day or later.

### Randomization

An independent statistician (Dr. Akiko Kada) composed tables of block randomization with three stratification factors using a computer-generated random allocation sequence. Stratification factors included sex, age (<40 or ≥40 years), and sense of life threat. This resulted in eight (2×2×2) different stratified tables of supplement numbers. Those tables were sent to an independent pharmacist (Professor Satoru Kobayashi); he securely kept the tables and prepared numbered supplements bottles according to the tables. Both the research team and participants will be blinded to randomization until the last participant has completed the protocol and the spreadsheets of all results are finalized. Stratification by sex, age, and sense of life threat has been justified by previous studies; the prevalence of PTSD and major depressive disorder were higher in women than in men [43,44]; and our previous cohort study of MVA survivors showed that sense of life threat was a strong predictor of subsequent PTSD [9]. Moreover, according to the National Health and Nutrition Survey on food group consumption by age group, the amount of meat products consumed such as pork and beef is higher for both sexes before age 40, but after this age the amount of seafood consumption increases. Also omega 3 fatty acid contents in red blood cells increase with age in the Japan population [45].

### Baseline assessment

Baseline data are collected on enrollment on the following items: general socio-demographics, smoking and alcohol consumption habits, detailed information about the accident, Injury Severity Score [46], Glasgow Coma Scale score [47], mild traumatic brain injury (i.e., concussion) defined by Hoge’s criteria [any of the following three conditions: losing consciousness (knocked out), being dazed, confused or seeing stars, or not remembering the injury] [48], type of accident, vital signs first assessed at the accident site and in the emergency room, regular medication, inhalation analgesic, past medical history, family history of psychopathology as determined in a structured interview, a sense of life threat during the accident as determined in the structured interview, the extent of memory regarding the accident as evaluated by the Memory of Traumatic Event Questionnaire [49], bodily pain at rest and on effort as measured subjectively using a visual analogue scale [50], performance status as defined by the Eastern Cooperative Oncology Group [51], and past history of traumatic event using the trauma checklist of the Clinician Administered PTSD Scale (CAPS) [52,53]. Other employed instruments include: the Peritraumatic Distress Inventory (PDI) [54,55], Conner-Davidson Resilience Scale (CD-RISC) [56], Buss-Perry Aggression Questionnaire (BAQ) [57], and the Food Frequency Questionnaire (FFQ) [58,59]. In a validation study, relatively high correlations between fatty acid intakes estimated from the FFQ and the corresponding serum phospholipid levels were observed as follows: EPA, r = 0.43 and r = 0.59 and DHA, r = 0.35 and r = 0.49 for crude value (g/day) and percentage of total fatty acid intake, respectively [60].

### Biological samples

#### Routine laboratory examination

Hematological parameters: white blood cell, erythrocyte, platelet, hemoglobin, and hematocrit.

Biochemistry: total protein, total albumin, total cholesterol, creatinine, BUN, GOT, GPT, γGTP, C-reactive protein.

#### Erythrocytes for fatty acid analysis

Seven milliliters of whole blood are obtained with ethylene-diamine tetraacetic acid in the emergency room (before ICU admission, baseline) and at the 3-month assessment for determination of fatty acids. Erythrocytes are washed twice with saline and frozen at −80°C until analysis. Total lipids are extracted according to the method of Bligh and Dyer [61]. Total phospholipid fractions are separated by thin-layer chromatography. After transmethylation with HCl–methanol, the fatty acid composition is analyzed by gas chromatography (GC-2014 Shimadzu Corporation, Kyoto, Japan) equipped with a DB-225 capillary column (length, 30 m; internal diameter, 0.25 mm; film 0.25 μm; J&M Scientific, Folsom, CA). The entire system is controlled using the gas chromatography software GC-solution version 2.3 (Shimadzu Corporation, Kyoto, Japan).

#### Serum for BDNF analysis

To assess the serum level of BDNF, 7–10 ml of blood are drawn at baseline, 1 month, and 3 months, and serum samples are stored in separate freezers at −80°C until analysis. We analyze all samples on the same day and under the same conditions. For the baseline blood sample, it is difficult to control for diurnal variation because the accident and admission can occur at any time; however, all blood samples at 1 month and 3 months are drawn between 10:00 and 16:00. Serum BDNF levels are measured using the BDNF Emax Immunoassay System kit (Promega, Madison, WI) according to the manufacturer’s instructions. The biochemist is blind to the diagnostic status of the participants. Other biomarkers such as neuropeptide-Y, dehydroepiandrosterone, activin A, and amino acids are also measured for comparison.

### Outcome measures

#### Psychiatric evaluations

The primary efficacy variable is the total score on the CAPS-DX [52,53]. Because there is potential overlap between organic and psychogenic symptoms with regard to PTSD, we will take care to ensure the symptoms are psychogenic in origin. Generally, if a symptom is better accounted for by an alternative explanation, we will categorize it as not being a psychological symptom [62]. Because injured patients involved in accidents often experience partial or full loss of traumatic memory, it can sometimes be difficult to differentiate organic from psychogenic amnesia on item 8 (psychogenic amnesia in criterion C3) of the CAPS. In this study, we adopt the standard manner of calculating all items for assessing PTSD symptoms as the primary outcome.

The secondary efficacy variables consist of the following: PTSD diagnosis as determined by CAPS-DX with all items including item 8, CAPS-DX total scores without item 8 when assessing PTSD symptoms [8], PTSD diagnosis as determined by CAPS-DX scores without item 8, total scores on the Montgomery-Asberg Depression Rating Scale (MADRS) [63,64], and major depressive disorder (MDD) as determined by the Mini International Neuropsychiatric Interview (MINI) [65,66]. Other secondary outcome measures are described elsewhere. Trained psychiatrists (YM, DN) conduct all of the face-to-face structured interviews for the evaluation of PTSD and other psychiatric morbidity at 1 and 3 months.

In this trial, participants are deemed to have partial PTSD if they fulfill two of three symptom criteria [B (re-experiencing), C (avoidance), or D (hyper-arousal)], and also the criteria A-1 (stressor), E (duration), or F (impairment). The inter-rater reliability of the diagnosis of PTSD and MDD was reported previously [67].

#### Psychological and psychophysiological evaluations

1. Impact of Event Scale-Revised [68]

The Impact of Event Scale-Revised (IES-R) is a self-reporting questionnaire about PTSD symptoms that was developed in the U.S. It is the most widely used measure internationally in all forms of disaster-area research. The IES-R is composed of 22 items on the three largest symptoms in the diagnostic criteria of PTSD, namely re-experiencing, avoidance, and increased physiological arousal. Respondents rate symptoms experienced in the previous week. The validity and reliability of the Japanese version of the IES-R has been confirmed [69].

2. Hospital Anxiety and Depression Scale [70]

The Hospital Anxiety and Depression Scale (HADS) includes a 7-item anxiety subscale and a 7-item depression subscale for assessing general psychological distress for the preceding week. Each item is rated on a scale of 0–3, with high scores denoting greater psychological distress. The validity and reliability of the Japanese version of the HADS has been confirmed [71].

3. The Medical Outcomes Study 36-Item Short Form Health Survey [72]

The Medical Outcomes Study 36-Item Short Form Health Survey (SF-36) is based on a conceptual model consisting of physical and mental health constructs, and is designed to measure perceived health status and daily functioning. It consists of 36 items scored in the following 8 domains: physical functioning, role-physical functioning, bodily pain, general health perception, vitality, social functioning, role-emotional functioning, and mental health [73]. The validity and reliability of the Japanese version of the SF-36 has been confirmed [74].

4. Connor-Davidson Resilience Scale [56]

The self-rating Connor-Davidson Resilience Scale (CD-RISC) comprises of 25 items, each rated on a 5-point scale (0–4), with high scores reflecting greater resilience. The Japanese version of the CD-RISC was translated by Nakajima and Kim and they are currently conducting a validation study.

5. Buss-Perry Aggression Questionnaire [57]

The Buss-Perry Aggression Questionnaire (BAQ) is a 29-item questionnaire where participants rank certain statements along a 5-point continuum from "extremely uncharacteristic of me" to "extremely characteristic of me." The scores are normalized on a scale of 0 to 1, with 1 being the highest level of aggression. It has 4 dimensions of aggression: physical aggression, verbal aggression, anger, and hostility. The validity and reliability of the Japanese version of the BAQ has been confirmed [75].

6. Physiological assessment

To avoid any confounding effect that the psychological and psychiatric assessment might have on autonomic response, we perform the physiologic assessment first [76]. The physiological data to be collected include heart rate, skin conductance, and continuous blood pressure while the patient is subjected to startling tones and idiographic trauma-related cues. The startling tones to be used in this study are essentially the same as those described in a previous study [77]. The idiographic script-driven imagery to be used is essentially the same as that described in a previous study [78]. The detailed experimental procedures were reported elsewhere [79].

### Data collection schedule

Data collection listed is conducted according to the appropriate timing and each aspect of the relevant information. The follow-up assessment schedule from baseline to 3 months is shown in Table 1.

**Table 1:** Outcome measures and assessments

	**ADMISSION**	**BASELINE**	**1 MONTH**	**3 MONTH**
Primary outcome				
CAPS			+	+
Secondary outcome				
MINI			+	+
MADRS			+	+
IES-R			+	+
HADS			+	+
SF-36			+	+
CD-RISC		+		+
BAQ		+		+
Physiologic experiment				+
Other measures				
Alcohol, smoking, FFQ		+		
PDI		+		
Life event after the accident			+	+
Serum	+		+	+
Erythrocyte	+			+

Table legend text: CAPS, Clinician-Administered PTSD Scale; MINI, Mini International Neuropsychiatric Interview; MADRS, Montgomery-Asberg Depression Rating Scale; IES-R, Impact of Event Scale revised; HADS, Hospital Anxiety and Depression Scale; SF-36, The 36-item short form of the MOS Questionnaire; CD-RISC, Conner-Davidson Resilience Scale; BAQ, Buss-Perry Aggression Questionnaire; FFQ, Food Frequency Questionnaire; PDI, Peritraumatic Distress Inventory.

#### Data collected at time of enrollment

1. Whole blood and serum: A blood sample is taken from patients when transferred to our emergency room and treated for the first time. If consent to participate in the study is provided, test samples are obtained by the Clinical Laboratory Department.

2. Demographic characteristics

3. Alcohol, smoking, food frequency questionnaire (FFQ)

4. Detailed information about accidental injury

5. Psychological response to accidental injury: sense of life threat, subjective severity of injury, MTEQ, PDI

6. Psychological assessment: CD-RISC, BAQ

7. Physical examination: Performance status, blood pressure at rest (right hand), heart rate, laboratory data

#### Data collected at 1 month after informed consent provided

1. Life events after accidental injury

2. Outpatient or inpatient treatment

3. Physical examination: Performance status, blood pressure at rest (right hand), heart rate, laboratory data

4. Psychiatric evaluation: MINI, CAPS, MADRS

5. Psychological response: IES-R, HADS

6. Serum

7. Adverse events

#### Data collected at 3 months after informed consent provided

1. Life events after accidental injury

2. Outpatient or inpatient treatment

3. Physical examination: Performance status, blood pressure at rest (right hand), heart rate, laboratory data

4. Psychiatric evaluation: MINI [65,66], CAPS [52,53], MADRS [63,64]

5. Psychological responses: IES-R, HADS, SF-36, CD-RISC [56], BAQ [57]

6. Physiological assessment

7. Whole blood and serum

8. Adverse events

9. Number of days of leave from work taken from the time of injury

### Procedure of psychiatric evaluation

Trained psychiatrists (YM, DN) conduct all of the face-to-face structured interviews for the evaluation of PTSD and other psychiatric morbidity at months 1 and 3. The primary method of evaluation is set as the face-to-face interview. However, conducting an interview via phone is also acceptable when an interview proves difficult to arrange. Clinical research coordinators notify participants of the upcoming follow-up survey no later than 5 days before a psychiatric evaluation. An outcome evaluator reserves a date for the next evaluation after the survey. When notifying participants via e-mail, an email template created specifically for this study is used and sent from an email account address designated for this study.

### Reporting of adverse events and protection of participants

An adverse event is defined as any unwanted or unintended sign, symptom, or disease seen in trial participants, regardless of the causal relationship with the study interventions. After enrollment, patients undergo a short physical examination and then complete the questionnaires and interviews. Safety issues concerning omega-3 fatty acid supplementation, including monitoring of vital signs and records of adverse events, are investigated at least twice a week during hospitalization. After discharge, safety issues are checked by telephone or email contact. When an adverse event is suspected, researchers and clinical research coordinators evaluate and record the event. Based on the following criteria, adverse events are evaluated for severity according to the following three levels.

1. Mild: Some symptoms or signs observed, but the participant can continue the trial without treatment.

2. Moderate: Some symptoms or signs observed, but the participant can continue the trial with treatment, such as reducing the dose of the test capsules or administering drugs.

3. Severe: Clinical status and daily activities are significantly affected, and discontinuation of the test capsules may be needed. Examples include headache, diarrhea, soft stool, and belching that are severe enough to reduce daily activities.

An association between the adverse event and test capsules is evaluated on four levels based on the following criteria. If it is judged there is “No association,” the reason for the judgment is also explained in the report.

1. Not related: Symptoms can be clearly explained by factors other than the test capsules.

2. Possibly related: Although the symptoms can be considered to be caused by factors other than the test capsules, a possible relationship between them cannot completely be ruled out.

3. Probably related: The symptoms are unlikely to be caused by factors other than the test capsules.

4. Related: Causal relationship with the test capsules is strongly suspected, due to a temporal relationship between test capsule administration and occurrence of the adverse event, a correlation between the dose of the test capsules and symptom severity, or when occurrence of the adverse event cannot be explained by factors other than the test capsules.

Serious adverse events include the following, regardless of the dose of the test capsules. Administration of the test capsules is discontinued upon the occurrence of any such event.

1. An event resulting in death

2. A life-threatening event (the participant is facing the possibility of death at the occurrence of the event)

3. An event requiring hospitalization or an extension of hospitalization to provide treatment

4. An event resulting in permanent or prominent disorder or functional failure

5. An event causing a subsequent congenital anomaly or congenital deficiency

6. Other events or reactions considered medically significant

Clinical research coordinators immediately contact the principal investigator (YM) and research committee when an adverse event or serious adverse event occurs. Any adverse event or serious adverse event is reported on a monitoring report and evaluated by the Data and Safety Monitoring Board regularly. A reported event gives the date of occurrence and description of the event. In addition, information is reported on the dose of the capsule, concomitant drug(s) and dosage, consumed supplements, types of food, and dates of start or discontinuation of the test capsules. The principal investigator gathers information about the occurrence of a serious event or event that may affect study continuation. The principal investigator also communicates and shares the information with the Data and Safety Monitoring Board independently from the other researchers and the provider of the test capsules. The ethics committee receives reports as needed.

### Failure to attend a hospital visit or failure to reach the participant or family

If a participant does not visit the hospital for a follow-up appointment, the clinical research coordinator calls the participant or their family and encourages a visit soon after confirming the reason for not visiting. If visiting is judged to be difficult, a phone interview is conducted instead. When contacting the participant, the coordinator confirms that the person talking over the phone is the participant himself or herself. When mailing materials, a confidential form is used to protect personal information.

### Outcomes

#### Primary outcome

Total score of the CAPS-DX at 3 months follow-up.

#### Secondary outcomes

1. Incidence of diagnosis of PTSD (full PTSD and partial PTSD) at 1 and 3 months follow-up

2. Total score of the CAPS-DX at 1 month follow-up

3. MADRS score at 1 and 3 months follow-up

4. Incidence of diagnosis of major depressive disorder evaluated by MINI at 1 and 3 months follow-up

5. Physiological response to script-driven imagery and audiovisual stimuli at 3 months follow-up

6. IES-R score at 1 and 3 months follow-up

7. HADS score at 1 and 3 months follow-up

8. SF-36 score at 1 and 3 months follow-up

9. CD-RISC score at baseline and 3 months follow-up

10. BAQ score at baseline and 3 months follow-up

11. Serum BDNF at baseline and 1 and 3 months follow-up

12. Number of days of leave from work taken from the time of injury

13. Dehydroepiandrosterone at 1 and 3 months follow-up

14. Neuropeptide Y at 1 and 3 months follow-up

15. Interleukin 1 beta at 1 and 3 months follow-up

16. Interleukin 6 at 1 and 3 months follow-up

17. Tumor necrosis factor alpha at 1 and 3 months follow-up

18. D-serine at 1 and 3 months follow-up

19. L-serine at 1 and 3 months follow-up

20. DL-serine at 1 and 3 months follow-up

21. Activin at 1 and 3 months follow-up

### Statistical analyses

All analyses are conducted according to the intention-to-treat principle.

#### Primary analysis

The primary purpose of this trial is to examine whether patients receiving omega-3 fatty acid supplementation after physical trauma score on average 10 points lower on the CAP scale at 3 months follow-up than those receiving placebo. Using analysis of covariance, mean score differences, 95% confidence intervals, and P values are calculated. Two-tailed tests will be used, and the α level will be set at 5%. If background factors are found that may influence prognosis even if the assignment code is blinded, those factors will be entered as covariates in the analysis.

#### Exploratory analysis

To complement the primary analyses, additional evaluation of secondary outcomes and sub-groups will be analyzed. The analysis will be exploratory, thus multiplicity will not be controlled. Evaluation with regression models in repeated measures as a mixed effect model will be conducted. Sensitivity analysis and imputed methods to missing variables will be used to examine the validity of the findings.

#### Estimation of sample size

Forty-nine cases are required, given that the expected CAP score difference between the groups at 3 months is set as 10, with an α level of 0.05 (two-tailed), a β level of .10, and standard deviation of 15. Discontinuation and dropout are estimated to be around 30% of the participants, indicating that 70 cases are needed. Thus, the desired number of participants in each group is 70, with a total of 140 cases. However, if this trial becomes difficult to continue due to finances, man-power, or physical factors, there is a possibility that the accumulation of cases will be stopped before the desired number is achieved.

#### Time periods of the study

Study period: December 2008 through December 2013

Enrollment period: December 2008 through June 2013

Follow-up period: December 2008 through December 2013

### Ethical considerations

The present study protects the rights and welfare of participants in the spirit of ethical guidelines outlined under the Declaration of Helsinki. The study further respects the ethical principles of the Ministry of Health, Labour, and Welfare of Japan. Confidence is assured in the ethics, safety, scientific rigor, and reliability of the research. Personal information obtained in the course of the research is strictly secured to avoid external leaks. No special compensation is paid in the event of health damage directly related to the research. The research plan (2007–12) was deliberated upon and approved by the Ethics Committee of the National Disaster Medical Center on February 8, 2008.

### Criteria for discontinuing intervention, evaluation, and follow-up

The criteria for discontinuing intervention, evaluation, and follow-up with participants are set as follows.

#### Discontinuing intervention

Investigators discontinue the intervention if the participant refuses to continue the treatment with the test capsules or placebo, or if further intervention is difficult because concomitant drug administration is required medically. Investigators ask the participant whether he or she can participate in later evaluations and follow-up. The process for discontinuing registration is initiated and reasons for the discontinuation are fully reported. Later evaluations and follow-up are conducted as designed.

#### Discontinuing evaluation

Psychiatric evaluation is discontinued if a participant refuses to undergo evaluation. Investigators ask the participants whether they can participate in later evaluations and follow-up. The process for discontinuing registration is initiated and reasons for the discontinuation are fully reported. Later evaluations and follow-up are conducted as designed.

#### Discontinuing follow-up

Study participation is discontinued if a participant refuses to be followed up. Investigators undertake the process of discontinuing registration for follow-up and record the relevant details. Later evaluations and follow-up are not conducted.

#### Retraction of consent

When a participant retracts his or her consent, all intervention, evaluation, and follow-up is discontinued.

#### Deviation from the study protocol

When a participant deviates from the study protocol as noted in the following, a follow-up session is conducted as far as is possible: 1) capsules are not supplied or taken as scheduled, 2) documents necessary for evaluation are missing, and 3) continuing intervention is deemed difficult by the investigators for other reasons. Reasons for the deviation are recorded.

#### Discontinuation of the study

The study is discontinued in the event of any of the following.

1. The Data and Safety Monitoring Board recommends discontinuation of the study.

2. Serious adverse events and side-effects occur that make continuing the study difficult, and the Board recommends discontinuation of the study.

3. The principal investigator decides to discontinue the study.

### Data and safety monitoring

The data manager provides a report using a pre-determined template to the principal investigator and the Data and Safety Monitoring Board on a regular basis (once every 6 to 12 months). The Data and Safety Monitoring Board consists of the following three professionals with experience in clinical trials, who are not involved in this trial: Professor Yasuhiro Otomo (critical care medicine), Dr. Tetsuo Nakabayashi (regulatory science), and Professor Ataru Inagaki (psychopharmacology). The statistical analysis manager reviews the reports filed by the data manager in advance. Contents of the template include the following.

1. Study progress with case registration

2. Status of psychiatric evaluations and relevant issues

3. Occurrence of adverse events

4. Discussion of other relevant issues, problematic cases, and events

### Revision of the research plan and change of processes

The principal investigator will summon or e-mail investigators to discuss possible revisions to the research plan when the regular monitoring report indicates the presence of serious adverse events or events that may affect continuation of the study, thus suggesting safety problems in the study.

### Data access and responsibility

Drs. Yutaka Matsuoka and Daisuke Nishi have full access to all of the data in the study and take responsibility for the integrity of the data and the accuracy of analysis.

### Presentation of the trial results

The findings will be presented in medical journals and at academic conferences. The founding body will also be notified of the results. An application for a press release at the Press Club will be initiated if significant research results are obtained and the study is accepted for publication by an influential academic journal. Mainly the principal investigator is the corresponding author in all presentations, and the first author and co-authors will be determined according to their intellectual contributions. In addition, individuals who join the study after it was approved and work fully toward the implementation and progress of the study may obtain authorship with the approval of the principal investigator.

### Funding

This study was supported by CREST, the Japan Science and Technology Agency (YM). The funding body peer reviewed proposal documents which included digest version of this study protocol as part of the selection process. All of the supplements used in the study were supplied by Kentech Co, Ltd., Toyama, Japan. They had no role in the study design and conduct, in the collection, analysis and interpretation of the data, or in the preparation, review, and approval of the manuscript.

## Discussion

It is suggested that hippocampal neurogenesis may play an important role in the periodic clearance of hippocampal memory traces in contextual fear conditioning [36]. Thus, hippocampal neurogenesis is emerging as a possible mediator of the effect of omega-3 fatty acids on fear memory. Modulating adult hippocampal neurogenesis by omega-3 fatty acid supplementation may well then be a good target for the prevention of PTSD. Intervention with omega-3 fatty acid appears acceptable in broad clinical practice because of its convenience, empirical results in animal studies, and much less serious side effects than any psychotherapeutic drugs.

The TPOP study is designed to evaluate the efficacy of omega-3 fatty acid supplementation for PTSD prevention among injured patients. The open trial [38] provided promising support for our hypothesis: omega-3 fatty acid supplementation significantly attenuated PTSD symptoms at 3 months as a primary outcome, as compared with the hypothetical mean. We believe the results of the TPOP will be of importance: accidental injury occurs across the globe and omega-3 fatty acid supplementation, if found to be efficacious for preventing PTSD, could maintain the mental health of the injured.

## Abbreviations

BAQ: Buss-Perry Aggression Questionnaire; BDNF: Brain-derived neurotrophic factor; CAPS: Clinician-Administered PTSD Scale; CD-RISC: Connor-Davidson Resilience Scale; DHA: Docosahexaenoic acid; EPA: Eicosapentaenoic acid; FFQ: Food Frequency Questionnaire; HADS: Hospital Anxiety and Depression Scale; ICU: Intensive care unit;IES-R: Impact of Event Scale-Revised; MADRS: Montgomery-Asberg Depression Rating Scale; MDD: Major depressive disorder; MINI: Mini International Neuropsychiatric Interview; MVA: Motor vehicle accident;PTSD: Posttraumatic stress disorder; SF-36: The 36-item short form of the MOS Questionnaire; TPOP: Tachikawa Project for Prevention of PTSD with Polyunsaturated Fatty Acid

## Competing interests

Dr. Matsuoka has received research support from the Japan Science and Technology Agency, CREST and the Ministry of Health, Labor, and Welfare of Japan, an Intramural Research Grant for Neurological and Psychiatric Disorders from NCNP and lecture fees from Suntory Wellness Ltd, Eli Lilly Japan KK, and Otsuka Pharmaceutical Co., Ltd. Dr. Nishi has received research support from Toray Industries, Inc. and the Foundation for Total Health Promotion, and lecture fees from Qol Co., Ltd, DHA & EPA Association, and NTT DoCoMo, Inc. Dr. K. Hamazaki has received research support from the Japan Society for the Promotion of Science, an Intramural Research Grant for Neurological and Psychiatric Disorders from NCNP, the Tamura Foundation for Promotion of Science and Technology, and the Ichiro Kanehara Foundation for Promotion of Medical Sciences and Medical Care, and consultant fees from Polyene Project, Inc. and Otsuka Pharmaceutical Co., Ltd., and lecture fees from Nippon Suisan Kaisha, Ltd. Dr. T. Hamazaki has received research support from the Japan Society for the Promotion of Science, Open Research Center for Lipid Nutrition (Kinjo Gakuin University), and Nippon Suisan Kaisha, Ltd., and consultant fees from Polyene Project, Inc. and Otsuka Pharmaceutical Co., Ltd., and lecture fees from Mochida Pharmaceutical Co., Ltd. Dr. Matsumura has received research support from the Japan Society for the Promotion of Science and the 26th Research Grant in Medical and Health Science from Meiji Yasuda Life Foundation of Health and Welfare. All other authors declare that they have no competing interests with this work.

## Authors’ contributions

YM is a principal investigator. YM and DN conceived the study and drafted the original protocol. NY calculated sample size and decided the analytic strategy. YM, DN, NY, KH and TH participated in the refinements of the protocol. YM, DN, KH, and TH decided the content of the omega-3 fatty acid supplementation. KM decided the method of psychophysiological experiment, KM and HN managed the experiment. HN managed clinical research coordinators. YM and DN managed the enrolment procedure and overall regulation of the trial. KH managed the serum analysis. All authors read and approved the final manuscript.

## Authors’ information

Daisuke Nishi is now at the Department of Mental Health Policy and Evaluation, National Institute of Mental Health, National Centre of Neurology and Psychiatry.

Kenta Matsumura is now at the School of Mechanical Engineering, College of Science and Engineering, Kanazawa University.

Tomohito Hamazaki is an ex-board member of International Society for the Study of Fatty Acids and Lipids and is now at Toyama Jonan Onsen Daini Hospital.

## Pre-publication history

The pre-publication history for this paper can be accessed here:

http://www.biomedcentral.com/1471-244X/13/8/prepub

## References

[B1] Krug EG Sharma GK Lozano R The global burden of injuries Am J Public Health 2000 90 4 523 526 10754963 10.2105/ajph.90.4.523 PMC1446200

[B2] MacKenzie EJ Rivara FP Jurkovich GJ Nathens AB Frey KP Egleston BL Salkever DS Scharfstein DO A national evaluation of the effect of trauma-center care on mortality N Engl J Med 2006 354 4 366 378 10.1056/NEJMsa052049 16436768

[B3] Verger P Dab W Lamping DL Loze J-Y Deschaseaux-Voinet C Abenhaim L Rouillon F The psychological impact of terrorism: an epidemiologic study of posttraumatic stress disorder and associated factors in victims of the 1995–1996 bombings in France Am J Psychiatry 2004 161 8 1384 1389 10.1176/appi.ajp.161.8.1384 15285963

[B4] Shalev AY Freedman S Peri T Brandes D Sahar T Orr SP Pitman RK Prospective study of posttraumatic stress disorder and depression following trauma Am J Psychiatry 1998 155 5 630 637 9585714 10.1176/ajp.155.5.630

[B5] Schnyder U Moergeli H Klaghofer R Buddeberg C Incidence and prediction of posttraumatic stress disorder symptoms in severely injured accident victims Am J Psychiatry 2001 158 4 594 599 10.1176/appi.ajp.158.4.594 11282694

[B6] O'Donnell ML Creamer M Pattison P Atkin C Psychiatric morbidity following injury Am J Psychiatry 2004 161 3 507 514 10.1176/appi.ajp.161.3.507 14992977

[B7] Hamanaka S Asukai N Kamijo Y Hatta K Kishimoto J Miyaoka H Acute stress disorder and posttraumatic stress disorder symptoms among patients severely injured in motor vehicle accidents in Japan Gen Hosp Psychiatry 2006 28 3 234 241 10.1016/j.genhosppsych.2006.02.007 16675367

[B8] Hepp U Moergeli H Buchi S Bruchhaus-Steinert H Kraemer B Sensky T Schnyder U Post-traumatic stress disorder in serious accidental injury: 3-year follow-up study Br J Psychiatry 2008 192 5 376 383 10.1192/bjp.bp.106.030569 18450664

[B9] Matsuoka Y Nishi D Nakajima S Kim Y Homma M Otomo Y Incidence and prediction of psychiatric morbidity after a motor vehicle accident in japan: the tachikawa cohort of motor vehicle accident study Crit Care Med 2008 36 1 74 80 10.1097/01.CCM.0000291650.70816.D6 18090377

[B10] Bryant RA O'Donnell ML Creamer M McFarlane AC Clark CR Silove D The psychiatric sequelae of traumatic injury Am J Psychiatry 2010 167 3 312 320 10.1176/appi.ajp.2009.09050617 20048022

[B11] Schnyder U Wittmann L Friedrich-Perez J Hepp U Moergeli H Posttraumatic stress disorder following accidental injury: rule or exception in Switzerland? Psychother Psychosom 2008 77 2 111 118 10.1159/000112888 18230944

[B12] Matsuoka Y Nishi D Yonemoto N Nakajima S Kim Y Towards an explanation of inconsistent rates of posttraumatic stress disorder across different countries: infant mortality rate as a marker of social circumstances and basic population health Psychother Psychosom 2010 79 1 56 57 10.1159/000259418 19923876

[B13] Schnyder U Moergeli H Trentz O Klaghofer R Buddeberg C Prediction of psychiatric morbidity in severely injured accident victims at one-year follow-up Am J Respir Crit Care Med 2001 164 4 653 656 11520732 10.1164/ajrccm.164.4.2008087

[B14] Ehlers A Clark DM Hackmann A McManus F Fennell M Herbert C Mayou R A randomized controlled trial of cognitive therapy, a self-help booklet, and repeated assessments as early interventions for posttraumatic stress disorder Arch Gen Psychiatry 2003 60 10 1024 1032 10.1001/archpsyc.60.10.1024 14557148

[B15] Vaiva G Ducrocq F Jezequel K Averland B Lestavel P Brunet A Marmar CR Immediate treatment with propranolol decreases posttraumatic stress disorder two months after trauma Biol Psychiatry 2003 54 9 947 949 10.1016/S0006-3223(03)00412-8 14573324

[B16] Pitman RK Sanders KM Zusman RM Healy AR Cheema F Lasko NB Cahill L Orr SP Pilot study of secondary prevention of posttraumatic stress disorder with propranolol Biol Psychiatry 2002 51 2 189 192 10.1016/S0006-3223(01)01279-3 11822998

[B17] Nishi D Matsuoka Y Kawase E Nakajima S Kim Y Mental health service requirements in a Japanese medical centre emergency department Emerg Med J 2006 23 6 468 469 10.1136/emj.2005.029769 16714512 PMC2564348

[B18] Hibbeln JR Fish consumption and major depression Lancet 1998 351 9110 1213 9643729 10.1016/S0140-6736(05)79168-6

[B19] Hibbeln JR Seafood consumption, the DHA content of mothers' milk and prevalence rates of postpartum depression: a cross-national, ecological analysis J Affect Disord 2002 69 1–3 15 29 12103448 10.1016/s0165-0327(01)00374-3

[B20] Noaghiul S Hibbeln JR Cross-national comparisons of seafood consumption and rates of bipolar disorders Am J Psychiatry 2003 160 12 2222 2227 10.1176/appi.ajp.160.12.2222 14638594

[B21] Suzuki S Akechi T Kobayashi M Taniguchi K Goto K Sasaki S Tsugane S Nishiwaki Y Miyaoka H Uchitomi Y Daily omega-3 fatty acid intake and depression in Japanese patients with newly diagnosed lung cancer Br J Cancer 2004 90 4 787 793 10.1038/sj.bjc.6601621 14970854 PMC2410186

[B22] Freeman MP Hibbeln JR Wisner KL Davis JM Mischoulon D Peet M Keck PE Jr Marangell LB Richardson AJ Lake J Omega-3 fatty acids: evidence basis for treatment and future research in psychiatry J Clin Psychiatry 2006 67 12 1954 1967 10.4088/JCP.v67n1217 17194275

[B23] Ross BM Seguin J Sieswerda LE Omega-3 fatty acids as treatments for mental illness: which disorder and which fatty acid? Lipids Health Dis 2007 6 21 10.1186/1476-511X-6-21 17877810 PMC2071911

[B24] Lin PY Su KP A meta-analytic review of double-blind, placebo-controlled trials of antidepressant efficacy of omega-3 fatty acids J Clin Psychiatry 2007 68 7 1056 1061 10.4088/JCP.v68n0712 17685742

[B25] Martins JG EPA but not DHA appears to be responsible for the efficacy of omega-3 long chain polyunsaturated fatty acid supplementation in depression: evidence from a meta-analysis of randomized controlled trials J Am Coll Nutr 2009 28 5 525 542 20439549 10.1080/07315724.2009.10719785

[B26] Sublette ME Ellis SP Geant AL Mann JJ **Meta-analysis of the effects of eicosapentaenoic acid (EPA) in clinical trials in depression** J Clin Psychiatry 2011 72 12 1577 1584 10.4088/JCP.10m06634 21939614 PMC3534764

[B27] Martins JG Bentsen H Puri BK Eicosapentaenoic acid appears to be the key omega-3 fatty acid component associated with efficacy in major depressive disorder: a critique of bloch and hannestad and updated meta-analysis Mol Psychiatry 2012 17 12 1144 1149 10.1038/mp.2012.25 22488258

[B28] Beltz BS Tlusty MF Benton JL Sandeman DC Omega-3 fatty acids upregulate adult neurogenesis Neurosci Lett 2007 415 2 154 158 10.1016/j.neulet.2007.01.010 17240063 PMC1892224

[B29] Wu A Ying Z Gomez-Pinilla F Dietary omega-3 fatty acids normalize BDNF levels, reduce oxidative damage, and counteract learning disability after traumatic brain injury in rats J Neurotrauma 2004 21 10 1457 1467 10.1089/neu.2004.21.1457 15672635

[B30] Calderon F Kim HY Docosahexaenoic acid promotes neurite growth in hippocampal neurons J Neurochem 2004 90 4 979 988 10.1111/j.1471-4159.2004.02520.x 15287904

[B31] Kawakita E Hashimoto M Shido O Docosahexaenoic acid promotes neurogenesis in vitro and in vivo Neuroscience 2006 139 3 991 997 10.1016/j.neuroscience.2006.01.021 16527422

[B32] Venna VR Deplanque D Allet C Belarbi K Hamdane M Bordet R PUFA induce antidepressant-like effects in parallel to structural and molecular changes in the hippocampus Psychoneuroendocrinology 2009 34 2 199 211 10.1016/j.psyneuen.2008.08.025 18848400

[B33] Squire LR Memory and Brain 1987 Oxford: Oxford University Press

[B34] Feng R Rampon C Tang YP Shrom D Jin J Kyin M Sopher B Miller MW Ware CB Martin GM Deficient neurogenesis in forebrain-specific presenilin-1 knockout mice is associated with reduced clearance of hippocampal memory traces Neuron 2001 32 5 911 926 10.1016/S0896-6273(01)00523-2 11738035

[B35] Ressler KJ Mayberg HS Targeting abnormal neural circuits in mood and anxiety disorders: from the laboratory to the clinic Nat Neurosci 2007 10 9 1116 1124 10.1038/nn1944 17726478 PMC2444035

[B36] Kitamura T Saitoh Y Takashima N Murayama A Niibori Y Ageta H Sekiguchi M Sugiyama H Inokuchi K Adult neurogenesis modulates the hippocampus-dependent period of associative fear memory Cell 2009 139 4 814 827 10.1016/j.cell.2009.10.020 19914173

[B37] Matsuoka Y Clearance of fear memory from the hippocampus through neurogenesis by omega-3 fatty acids: a novel preventive strategy for posttraumatic stress disorder? Biopsychosoc Med 2011 5 3 10.1186/1751-0759-5-3 21303552 PMC3045887

[B38] Matsuoka Y Nishi D Yonemoto N Hamazaki K Hashimoto K Hamazaki T Omega-3 fatty acids for secondary prevention of posttraumatic stress disorder after accidental injury: an open-label pilot study J Clin Psychopharmacol 2010 30 2 217 219 10.1097/JCP.0b013e3181d48830 20520307

[B39] Matsuoka Y Nishi D Yonemoto N Hamazaki K Hamazaki T Hashimoto K Potential role of brain-derived neurotrophic factor in omega-3 fatty acid supplementation to prevent posttraumatic distress after accidental injury: an open-label pilot study Psychother Psychosom 2011 80 5 310 312 10.1159/000322980 21720194

[B40] Folstein MF Folstein SE McHugh PR "Mini-mental state". A practical method for grading the cognitive state of patients for the clinician J Psychiatr Res 1975 12 3 189 198 10.1016/0022-3956(75)90026-6 1202204

[B41] Hamazaki T Sawazaki S Itomura M Asaoka E Nagao Y Nishimura N Yazawa K Kuwamori T Kobayashi M The effect of docosahexaenoic acid on aggression in young adults. A placebo-controlled double-blind study J Clin Invest 1996 97 4 1129 1133 10.1172/JCI118507 8613538 PMC507162

[B42] Sawazaki S Hamazaki T Yazawa K Kobayashi M The effect of docosahexaenoic acid on plasma catecholamine concentrations and glucose tolerance during long-lasting psychological stress: a double-blind placebo-controlled study J Nutr Sci Vitaminol 1999 45 5 655 665 10.3177/jnsv.45.655 10683816

[B43] Brewin CR Andrews B Valentine JD Meta-analysis of risk factors for posttraumatic stress disorder in trauma-exposed adults J Consult Clin Psychol 2000 68 5 748 766 11068961 10.1037//0022-006x.68.5.748

[B44] Ozer EJ Best SR Lipsey TL Weiss DS Predictors of posttraumatic stress disorder and symptoms in adults: a meta-analysis Psychol Bull 2003 129 1 52 73 12555794 10.1037/0033-2909.129.1.52

[B45] Itomura M Fujioka S Hamazaki K Kobayashi K Nagasawa T Sawazaki S Kirihara Y Hamazaki T Factors influencing EPA+DHA levels in red blood cells in Japan In Vivo 2008 22 1 131 135 18396795

[B46] Baker SP O'Neill B The injury severity score: an update J Trauma 1976 16 11 882 885 10.1097/00005373-197611000-00006 994270

[B47] Teasdale G Jennett B Assessment of coma and impaired consciousness. A practical scale Lancet 1974 2 7872 81 84 4136544 10.1016/s0140-6736(74)91639-0

[B48] Hoge CW McGurk D Thomas JL Cox AL Engel CC Castro CA Mild traumatic brain injury in U.S. soldiers returning from iraq N Engl J Med 2008 358 5 453 463 10.1056/NEJMoa072972 18234750

[B49] Gil S Caspi Y Ben-Ari IZ Koren D Klein E Does memory of a traumatic event increase the risk for posttraumatic stress disorder in patients with traumatic brain injury? a prospective study Am J Psychiatry 2005 162 5 963 969 10.1176/appi.ajp.162.5.963 15863799

[B50] Scott J Huskisson EC Graphic representation of pain Pain 1976 2 2 175 184 10.1016/0304-3959(76)90113-5 1026900

[B51] Oken MM Creech RH Tormey DC Horton J Davis TE McFadden ET Carbone PP Toxicity and response criteria of the Eastern Cooperative Oncology Group Am J Clin Oncol 1982 5 6 649 655 10.1097/00000421-198212000-00014 7165009

[B52] Blake DD Weathers FW Nagy LM Kaloupek DG Gusman FD Charney DS Keane TM The development of a clinician-administered PTSD scale J Trauma Stress 1995 8 1 75 90 10.1002/jts.2490080106 7712061

[B53] Asukai N Hirohata S Kato H Konishi T Psychometric properties of the japanese-language version of the clinician-administered PTSD scale for DSM-IV (in japanese) Jpn J Traumatic Stress 2003 1 1 47 53

[B54] Brunet A Weiss DS Metzler TJ Best SR Neylan TC Rogers C Fagan J Marmar CR The peritraumatic distress inventory: a proposed measure of PTSD criterion A2 Am J Psychiatry 2001 158 9 1480 1485 10.1176/appi.ajp.158.9.1480 11532735

[B55] Nishi D Matsuoka Y Noguchi H Sakuma K Yonemoto N Yanagita T Homma M Kanba S Kim Y Reliability and validity of the japanese version of the peritraumatic distress inventory Gen Hosp Psychiatry 2009 31 1 75 79 10.1016/j.genhosppsych.2008.09.002 19134513

[B56] Connor KM Davidson JR Development of a new resilience scale: the connor-davidson resilience scale (CD-RISC) Depress Anxiety 2003 18 2 76 82 10.1002/da.10113 12964174

[B57] Buss AH Perry M The aggression questionnaire J Pers Soc Psychol 1992 63 3 452 459 1403624 10.1037//0022-3514.63.3.452

[B58] Tsubono Y Takamori S Kobayashi M Takahashi T Iwase Y Iitoi Y Akabane M Yamaguchi M Tsugane S A data-based approach for designing a semiquantitative food frequency questionnaire for a population-based prospective study in Japan J Epidemiol 1996 6 1 45 53 10.2188/jea.6.45 8795957

[B59] Tsugane S Sasaki S Kobayashi M Tsubono Y Akabane M Validity and reproducibility of the self-administered food frequency questionnaire in the JPHC study cohort I: study design, conduct and participant profiles J Epidemiol 2003 13 1 Suppl S2 S12 12701628 10.2188/jea.13.1sup_2 PMC9767693

[B60] Kobayashi M Sasaki S Kawabata T Hasegawa K Tsugane S Validity of a self-administered food frequency questionnaire used in the 5-year follow-up survey of the JPHC study cohort I to assess fatty acid intake: comparison with dietary records and serum phospholipid level J Epidemiol 2003 13 1 Suppl S64 S81 12701633 10.2188/jea.13.1sup_64 PMC9767695

[B61] Bligh EG Dyer WJ A rapid method of total lipid extraction and purification Can J Biochem Physiol 1959 37 8 911 917 13671378 10.1139/o59-099

[B62] O'Donnell ML Creamer M Bryant RA Schnyder U Shalev A Posttraumatic disorders following injury: an empirical and methodological review Clin Psychol Rev 2003 23 4 587 603 10.1016/S0272-7358(03)00036-9 12788111

[B63] Montgomery SA Asberg M A new depression scale designed to be sensitive to change Br J Psychiatry 1979 134 382 389 10.1192/bjp.134.4.382 444788

[B64] Takahashi N Tomita K Higuchi T Inada T The inter-rater reliability of the japanese version of the montgomery-asberg depression rating scale (MADRS) using a structured interview guide for MADRS (SIGMA) Hum Psychopharmacol 2004 19 3 187 192 10.1002/hup.576 15079853

[B65] Sheehan DV Lecrubier Y Sheehan KH Amorim P Janavs J Weiller E Hergueta T Baker R Dunbar GC The Mini-International Neuropsychiatric Interview (M.I.N.I.): the development and validation of a structured diagnostic psychiatric interview for DSM-IV and ICD-10 J Clin Psychiatry 1998 59 20 22 33 quiz 34–57 9881538

[B66] Otsubo T Tanaka K Koda R Shinoda J Sano N Tanaka S Aoyama H Mimura M Kamijima K Reliability and validity of japanese version of the mini-international neuropsychiatric interview Psychiatry Clin Neurosci 2005 59 5 517 526 10.1111/j.1440-1819.2005.01408.x 16194252

[B67] Matsuoka Y Nishi D Nakajima S Yonemoto N Hashimoto K Noguchi H Homma M Otomo Y Kim Y The Tachikawa cohort of motor vehicle accident study investigating psychological distress: design, methods and cohort profiles Soc Psychiatry Psychiatr Epidemiol 2009 44 4 341 10.1007/s00127-009-0496-4 18818856

[B68] Weiss DS Marmar CR Wilson JP, Keane TM The Impact of Event Scale Revised Assessing psychological trauma and PTSD 1997 New York: The Guilford Press 399 411

[B69] Asukai N Kato H Kawamura N Kim Y Yamamoto K Kishimoto J Miyake Y Nishizono-Maher A Reliability and validity of the Japanese-language version of the impact of event scale-revised (IES-R-J): four studies of different traumatic events J Nerv Ment Dis 2002 190 3 175 182 10.1097/00005053-200203000-00006 11923652

[B70] Zigmond AS Snaith RP The hospital anxiety and depression scale Acta Psychiatr Scand 1983 67 6 361 370 10.1111/j.1600-0447.1983.tb09716.x 6880820

[B71] Kugaya A Akechi T Okuyama T Okamura H Uchitomi Y Screening for psychological distress in japanese cancer patients Jpn J Clin Oncol 1998 28 5 333 338 10.1093/jjco/28.5.333 9703862

[B72] McHorney CA Ware JE Jr Raczek AE The MOS 36-item short-form health survey (SF-36): II. Psychometric and clinical tests of validity in measuring physical and mental health constructs Med Care 1993 31 3 247 263 10.1097/00005650-199303000-00006 8450681

[B73] Ware JE Snow KK Kosinski M Gandek B SF-36 health survey manual and interpretation guide 1993 Boston: New England Medical Center

[B74] Fukuhara S Bito S Green J Hsiao A Kurokawa K Translation, adaptation, and validation of the SF-36 Health Survey for use in Japan J Clin Epidemiol 1998 51 11 1037 1044 10.1016/S0895-4356(98)00095-X 9817121

[B75] Ando A Soga S Yamasaki K Shimai S Shimada H Utsuki N Oashi O Sakai A Development of the japanese version of the buss-perry aggression questionnaire (BAQ) Shinrigaku kenkyu: The Japanese journal of psychology 1999 70 5 384 392 10.4992/jjpsy.70.384 10756586

[B76] Tucker PM Pfefferbaum B North CS Kent A Burgin CE Parker DE Hossain A Jeon-Slaughter H Trautman RP Physiologic reactivity despite emotional resilience several years after direct exposure to terrorism Am J Psychiatry 2007 164 2 230 235 10.1176/appi.ajp.164.2.230 17267785

[B77] Orr SP Lasko NB Shalev AY Pitman RK Physiologic responses to loud tones in Vietnam veterans with posttraumatic stress disorder J Abnorm Psychol 1995 104 1 75 82 7897056 10.1037//0021-843x.104.1.75

[B78] Pitman RK Orr SP Forgue DF de Jong JB Claiborn JM Psychophysiologic assessment of posttraumatic stress disorder imagery in Vietnam combat veterans Arch Gen Psychiatry 1987 44 11 970 975 10.1001/archpsyc.1987.01800230050009 3675137

[B79] Matsumura K Noguchi H Nishi D Matsuoka Y Effects of omega-3 fatty acids on the psychophysiologoical assessment for secondary prevention of posttraumatic stress disorder: and open-label pilot study Global Journal of Health Science 2012 4 1 3 9 22980098 10.5539/gjhs.v4n1p3 PMC4777038

